# Is there a gap between health education content and practice toward schistosomiasis prevention among schoolchildren along the shores of Lake Victoria in Kenya?

**DOI:** 10.1371/journal.pntd.0007572

**Published:** 2019-08-19

**Authors:** Rie Takeuchi, Sammy M. Njenga, Yoshio Ichinose, Satoshi Kaneko, Crystal A. Estrada, Jun Kobayashi

**Affiliations:** 1 Kenya Research Station, Institute of Tropical Medicine, Nagasaki University, Nairobi, Kenya; 2 Eastern and Southern Africa Centre of International Parasite Control, Kenya Medical Research Institute, Nairobi, Kenya; 3 Department of Eco-epidemiology, Institute of Tropical Medicine, Nagasaki University, Nagasaki, Japan; 4 Department of Global Health, Graduate School of Health Sciences, University of the Ryukyus, Okinawa, Japan; Faculty of Science, Ain Shams University (ASU), EGYPT

## Abstract

Despite provision of preventive measures against schistosomiasis such as mass drug administration (MDA), the prevalence of *Schistosoma mansoni* remains high in communities living near Lake Victoria. This study aimed to analyse the status of schistosomiasis, including its prevalence, health education, knowledge, attitudes, and practices (KAP) among pupils, and water use in schools in Mbita situated along the shores of Lake Victoria. Four primary schools were selected as target schools and pupils in classes six and seven were recruited as study participants. The prevalence of *S*. *mansoni* was examined by Kato-Katz method. Simultaneously, a KAP survey toward schistosomiasis was conducted among the pupils. Health education contents were extracted from textbooks. All primary schools in the study site were surveyed regarding how each secured water used for daily school life. The prevalence of *S*. *mansoni* was 56% and 36% in 2015 and 2016, respectively. 60–70% of pupils chose a correct answer for the mode of transmission. More than 70% of pupils answered that bathing in Lake Victoria causes *Schistosoma* infection; however, more than 70% of pupils bathed in Lake Victoria sometimes or every day. According to the science textbook, “avoiding contact with contaminated water” is the way to prevent schistosomiasis; however, 66% of schools asked pupils to bring water from Lake Victoria. The prevalence of *S*. *mansoni* among pupils remains high. Schoolchildren are taught to avoid contact with contaminated water but are often asked to fetch water from the lake. From the school health viewpoint, health education that reflects the social and cultural context of the community in the contents and teaching methods are needed. In addition to this, provision of sanitation infrastructure is needed. A comprehensive and innovative approach which harmonises central and local governments and other stakeholders, as well as community is important to prevent schistosomiasis.

## Author summary

According to WHO, it is estimated that at least 206 million people required preventive treatment for schistosomiasis in 2016 and of those at least 90% live in Africa. Moreover, school-age children are most affected by this worm. In Kenya, despite preventive measures against schistosomiasis such as mass drug administration (MDA) in primary schools, it remains a major public health problem, especially along the shores of Lake Victoria. Our study revealed that the prevalence of *Schistosoma mansoni* was high among pupils despite their knowledge about schistosomiasis transmission. This can be attributed to lack of safe water supply and latrines. A comprehensive approach which harmonises central and local governments and other stakeholders, as well as community is needed to prevent schistosomiasis. For example, the combination of health education (in which schools play as focal point) with provision of water infrastructure, encouragement to use and construct toilets in both schools and community/households, snail control, and MDA with monitoring can be employed to control and prevent schistosomiasis. From the school health viewpoint, it is necessary to develop health education contents and teaching methods that reflect the social and cultural context of the community in order to improve their behaviour and change the social norm.

## Introduction

Schistosomiasis is a parasitic disease caused by *Schistosoma* species. The parasite infects humans through skin penetration during contact with water that is contaminated with schistosome cercariae. Schistosomiasis is listed as a neglected tropical disease (NTD) by the World Health Organization (WHO); it is endemic in subtropical and tropical areas with most of the burden in sub-Saharan Africa [1]. In 2012, WHO reported that an estimated 3,179,000 years lived with disability (YLDs) were attributable to schistosomiasis [2]. High prevalence and intensity are observed among school-age children, adolescents, and young adults [3,4]. Since 1950, the World Health Assembly (WHA) has repeatedly discussed control and elimination of schistosomiasis and made several resolutions [5–9], and various measures, such as snail control, mass treatment, sanitary methods, and health education, have been conducted by several organizations worldwide [10]. In 2002, WHO recommended three components for schistosomiasis control in school: drug treatment, sanitary improvement, and health education. All three components interrupt the life cycle of schistosomes [11]. To determine the success of a control programme, a knowledge, attitude, and practice (KAP) survey which can collect information on what is known, believed, and done toward the topic of interest among the target population is useful [12]. Moreover, it is essential to know the social, cultural, and behavioural determinants which can fill the gap between knowledge and practice [13]. So far, several studies on KAP toward schistosomiasis have been conducted in various African countries among various participants. Most of them found that both adult and child participants did not have enough knowledge and good practices, and this reality may disrupt schistosomiasis control [14–18].

In Kenya, urinary and intestinal schistosomiasis due to *S*. *haematobium and S*. *mansoni* infection are observed and highly endemic in Coast region and Western Kenya, respectively [19]. The Government of Kenya launched the National School-Based Deworming Programme (NSBDP) in 2009 through a collaboration between the Ministry of Education and Ministry of Health [20]. In terms of sanitary improvement, water, sanitation and hygiene (WASH) activities in schools and/or communities have been conducted in several parts of Kenya by several organisations [21–24]. Despite implementation of preventive interventions such as mass drug administration (MDA) and WASH, the prevalence of *S*. *mansoni* in Mbita Sub-county along the shores of Lake Victoria has remained high [25–27]. This study aimed to analyse the status quo of schistosomiasis from a school health viewpoint. Specifically, this study described the prevalence, knowledge, attitude, and practice toward schistosomiasis among school children, health message through education, water use in primary schools enrolled in a school health project funded by Japan International Cooperation Agency (JICA), and the gap between messages from current preventive measures and actual practices.

## Methods

### Ethics statement

This study was approved by the Scientific and Ethics Review Unit, Kenya Medical Research Institute (SSC Protocol No. 2916). Permission for research activities in schools was given by the local education office. Written assent and written consent from pupils and their guardians, respectively, were obtained before commencement of data collection.

### Study site

**Fig 1:**
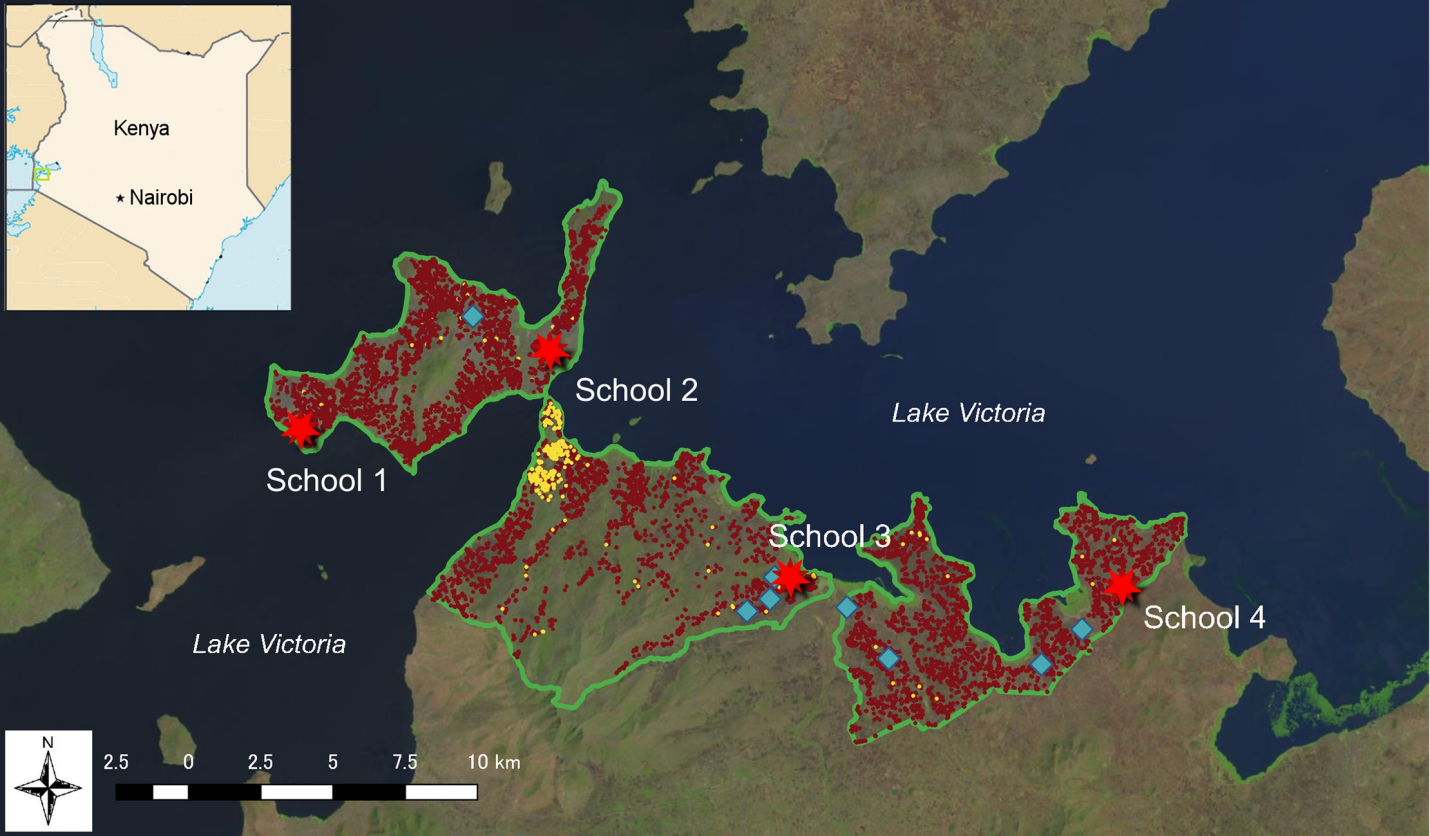
Study site and target schools. A green rectangle in the national map indicates the position of the area shown in the main map frame. Red stars in the main frame indicate target schools. Blue rhombuses indicate boreholes. Brown small dots indicate households that do not have piped water. Cream small dots indicate households that have piped water. **Base map data was downloaded from USGS LandsatLook https://landsatlook.usgs.gov/viewer.html Kenya map image was downloaded from CIA World Factbook https://www.cia.gov/library/publications/the-worldfactbook/graphics/maps/large/ke-map.gif**.

This study was conducted in four locations along the shores of Lake Victoria, in Mbita Sub-county, Homa Bay County, Kenya. The population of the study site in 2015 was 62,954, with fishery in Lake Victoria being the main source of income. In 2016, 3.3% of households had tap water and according to the Health and Demographic Surveillance System (HDSS) initiated by the NUITM-KEMRI project (Institute of Tropical Medicine, Nagasaki University, Japan and Kenya Medical Research Institute, Kenya), there were nine functional boreholes available in the area. The distribution of households having tap water, functional borehole, and target schools are shown in Fig 1. According to a school health project which has been conducted in the same area by the Institute of Tropical Medicine, Nagasaki University and funded by JICA since September 2012, there were 93 primary schools, including both public and private, complete and incomplete in the study area in 2016.

### Study participants

The sample size was calculated using Epi Info 7 (CDC, USA). Using the sample size calculation method for a population survey with four clusters, the calculated sample size was 67 for each cluster, giving a total sample size of 268.

One school from each location in the study area, giving a total of four schools, were randomly selected through the following procedure: first, schools which already received or were currently receiving any intervention from other organizations/groups were excluded. The remaining schools were then assigned individual numbers according to locations, with the numbers for each location selected randomly through draw lots. To examine the prevalence of *S*. *mansoni* and *S*. *haematobium* and KAP toward schistosomiasis, pupils in class six and class seven were recruited to participate in this study. All 93 schools in the study area were invited to provide information on how they obtained water being used in schools.

### Data collection

The presence of *S*. *mansoni* and *S*. *haematobium* infection was examined by Kato-Katz and urine filtration methods, respectively, by skilled laboratory technicians [28,29]. To increase sensitivity of the Kato-Katz method, stool samples collected over three consecutive days were examined for *S*. *mansoni* eggs. The presence of soil-transmitted helminths (STHs) was also recorded. Urine samples were collected once. Stool samples were collected in the morning, from 7:30 to 8:00 a.m. before class would start, and urine samples were collected during school morning break which was scheduled from 11:00 a.m. to 11:30 a.m. Two slides were prepared from each sample and examined by two independent microscopists to detect *S*. *mansoni* or *S*. *haematobium* eggs. Intensity of infection was calculated as the number of eggs per gram of feces (epg) or per 10 ml of urine and categorised as light (1–99 epg), moderate (100–399 epg), or heavy (≥ 400 epg) according to WHO guideline [30]. Treatment with praziquantel were given to all schistosomiasis-positive pupils who participated in the study.

A schistosomiasis KAP survey was conducted among the pupils using a paper-based, close-ended questionnaire. The questions that were asked included cause of infection, symptoms, prevention, and risk behaviours. Contents related to health education against schistosomiasis was extracted from primary school science textbooks by the researchers. Additionally, secondary data obtained from teacher interviews for a previous school health project were also used in the present study. Information that were obtained from the teacher interviews include how the water for daily school use such as drinking, washing hands, cleaning facilities, and school gardening was acquired. The stool and urine examinations and KAP survey among pupils were conducted twice—in October 2015 and September 2016, while the contents extraction of health education textbooks was carried out between October 2015 to September 2016. Finally, the survey on water use in schools was done in October 2016.

### Data analysis

Results of the laboratory exam and KAP survey in 2015 and 2016 were described using descriptive statistics while logistic regression modelling was used to explore the association between attitude and practice and to identify the potential risk factors of *Schistosoma* infection. 2015 and 2016 data were integrated for analysis of association and risk factors. All statistical analyses were performed using Stata 13.0 computer package (StataCorp., USA).

## Results

### Enrolment and characteristics of participants

Table 1 shows the demographic characteristics of the study participants. 274 pupils (165 boys and 109 girls) participated in 2015. Similarly, 274 (145 boys and 129 girls) pupils also participated in the study in 2016 (Table 1). Mean age in year were 13.6 ± 1.2 in 2015 and 13.4 ± 1.1 in 2016.

**Table 1:** Number of study participants.

	2015			2016		
	N	Boys: n (%)	Girls: n (%)	N	Boys: n (%)	Girls: n (%)
School 1	73	41 (56)	32 (44)	70	34 (49)	36 (51)
School 2	43	29 (67)	14 (33)	53	27 (51)	26 (49)
School 3	77	48 (62)	29 (38)	72	39 (54)	33 (46)
School 4	81	47 (58)	34 (42)	79	45 (57)	34 (43)
Total	274	165 (60)	109 (40)	274	145 (53)	129 (47)

### Prevalence and intensity of *S*. *mansoni*, *S*. *haematobium* and STHs

A total of 218 (133 boys and 85 girls) and 219 (118 boys and 101 girls) pupils were successfully examined for *S*. *mansoni* and STH on three consecutive days in 2015 and 2016, respectively. The prevalence of *S*. *mansoni* infection was 56.4% in 2015 and 35.6% in 2016. The percentage of pupils with light, moderate, and heavy infection were 32.6%, 16.5%, and 7.3% in 2015 and 23.3%, 9.6%, and 2.7% in 2016, respectively (Table 2). The prevalence of STH infections was low both in 2015 and 2016; specifically, the prevalence of hookworm, *Ascaris lumbricoides*, and *Trichuris trichiura* were 2.8%, 0.5%, and 0.9% in 2015. Only *A*. *lumbricoides* was detected in 2016 at 0.5% prevalence. No *S*. *haematobium* infection was detected.

**Table 2:** Rate and intensity of *Schistosoma mansoni* infection among pupils.

	2015					2016				
	N	Positive: n (%)	Light: n (%)	Moderate: n (%)	Heavy: n (%)	N	Positive: n (%)	Light: n (%)	Moderate: n (%)	Heavy: n (%)
Overall	218	123 (56.4)	71 (32.6)	36 (16.5)	16 (7.3)	219	78 (35.6)	51 (23.3)	21 (9.6)	6 (2.7)
By school										
School 1	59	38 (64.4)	25 (42.4)	7 (11.9)	6 (10.2)	62	29 (46.8)	21 (33.9)	7 (11.3)	1 (1.6)
School 2	39	37 (94.9)	19 (48.7)	12 (30.8)	6 (15.4)	48	27 (56.3)	14 (29.2)	8 (16.7)	5 (10.4)
School 3	52	2 (3.9)	2 (3.9)	0 (0)	0 (0)	51	0 (0)	0 (0)	0 (0)	0 (0)
School 4	68	46 (67.7)	25 (36.8)	17 (25.0)	4 (5.9)	58	22 (37.9)	16 (27.6)	6 (10.3)	0 (0)

### KAP on schistosomiasis

Table 3 summarizes the KAP results. More than 75% of pupils in both 2015 and 2016 answered that blood in urine and blood in stool were main signs of schistosomiasis. However, more than half of the pupils wrongly chose “blood in urine” as a main sign of dominant schistosomiasis in Mbita (data is available only for 2016). Nearly 60–70% of pupils in 2015 and 2016 correctly answered “contact with contaminated water” as a transmission mode. In terms of prevention, “Using protective boots and gloves when we contact with water from lake”, “avoiding contact with contaminated water” and “using toilet for urination/defecation” were the top three major answers for both years. Pupils cited schoolteachers (more than 90%) or hospital/health centre (about 80%) as their main sources of information. In terms of attitude, about half of pupils chose “strongly disagree” or “disagree” to having the possibility to get infected with schistosomiasis in daily life in both years. On the other hand, more than 70% of pupils “strongly agreed” or “agreed” to possibly getting infected with schistosomiasis by bathing/fetching water/washing clothes or utensils at Lake Victoria. Attitude toward infection opportunity during daily life and activities at the lake were statistically different (*p* < 0.001 in 2016 by χ^2^ test). In terms of practice, more than 70%, 80%, and 70% of pupils bathed, fetched water and washed clothes/utensils, respectively at Lake Victoria every day or sometimes in both years. When asked about sanitation habits, about 85% of the pupils reported that they regularly use toilet while in school, but the figure statistically decreased to about 67% while at home in both years (*p* = 0.004 in 2015, *p* < 0.001 in 2016 by χ^2^ test).

**Table 3:** Results of knowledge, attitude and practice towards schistosomiasis by year (N_15_ = 264, N_16_ = 263).

	2015 n (%)	2016 n (%)
**Knowledge**		
Signs and symptoms (multiple answer)		
Abdominal pain	93 (35.2)	91 (34.6)
Diarrhoea	86 (32.6)	105 (40.0)
Blood in urine	234 (88.6)	232 (88.2)
Blood in stool	202 (76.5)	226 (85.9)
Severe fever	77 (29.2)	89 (33.8)
Itching	88 (33.3)	85 (32.3)
Transmission* (N_15_ = 185, N_16_ = 229)		
Eating raw/undercooked fish/meat	7 (3.8)	7 (3.1)
Eating unwashed fresh vegetables	10 (5.4)	17 (7.4)
Contact with contaminated water	110 (59.5)	159 (69.4)
Walking barefoot on contaminated soil	58 (31.3)	46 (20.1)
Prevention (multiple answer)		
Cooking fish/meat well	50 (18.9)	33 (12.6)
Washing fresh vegetables	69 (26.1)	57 (21.7)
Avoiding contact with contaminated water	149 (56.4)	221 (84.0)
Using toilet for urination/defecation	146 (55.3)	129 (49.1)
Wearing sandals	84 (31.8)	99 (37.6)
Using protective boots and gloves when we contact with water from lake	196 (74.2)	181 (68.8)
Washing hands before eating	101 (38.3)	88 (33.5)
Washing hands after using toilets	108 (40.9)	117 (44.5)
Source of information (multiple answer)		
Hospital/health centre	202 (76.5)	211 (80.2)
School teachers	247 (93.6)	242 (92.0)
Family	80 (30.3)	99 (37.6)
Friends/neighbours	77 (29.2)	96 (36.5)
CHV	27 (10.2)	46 (17.5)
Main sign of bilharzia in Mbita* (N_16_ = 245)		
I do not know.	No data	9 (3.7)
Blood in urine		139 (56.7)
Blood in stool		97 (39.6)
**Attitude**		
Having an opportunity to be infected with bilharzia		
Strongly disagree	97 (36.7)	101 (38.4)
Disagree	40 (15.1)	32 (12.2)
Neutral	15 (5.7)	15 (5.7)
Agree	44 (16.7)	21 (8.0)
Strongly agree	68 (25.8)	94 (35.7)
Having an opportunity to be infected with bilharzia by bathing/fetching water/washing clothes or utensils at Lake Victoria	No data	
Strongly disagree		42 (16.0)
Disagree		15 (5.7)
Neutral		13 (4.9)
Agree		19 (7.2)
Strongly agree		174 (66.2)
**Practice**		
Bathing at Lake Victoria		
Everyday	148 (56.1)	132 (50.2)
Sometimes	61 (23.1)	57 (21.7)
Rarely	16 (6.0)	29 (11.0)
Never	39 (14.8)	45 (17.1)
Fetching water from Lake Victoria		
Everyday	139 (52.7)	150 (57.0)
Sometimes	84 (31.8)	74 (28.1)
Rarely	22 (8.3)	22 (8.4)
Never	19 (7.2)	17 (6.5)
Washing clothes/utensils at Lake Victoria		
Everyday	86 (32.6)	88 (33.5)
Sometimes	104 (39.4)	96 (36.5)
Rarely	22 (8.3)	30 (11.4)
Never	52 (19.7)	49 (18.6)
Using toilet at school		
Not at all	10 (3.8)	5 (1.9)
Sometimes	31 (11.7)	25 (9.5)
Every time	223 (84.5)	233 (88.6)
Using toilet at home		
Do not have a toilet at home	20 (7.6)	15 (5.7)
Not at all	20 (7.6)	19 (7.2)
Sometimes	48 (18.2)	52 (19.8)
Every time	176 (66.6)	177 (67.3)
Playing (swimming) at Lake Victoria	No data	
Everyday		58 (22.0)
Sometimes		93 (35.4)
Rarely		30 (11.4)
Never		82 (31.2)

*Participants who selected multiple answers were excluded.

Risk factors for schistosomiasis were bathing in the lake (OR for everyday = 2.66, 95%CI: 1.49–4.74), fetching water from the lake (OR for everyday = 2.34, 95%CI: 1.05–5.22), washing clothes/utensils in the lake (OR for everyday = 3.30, 95%CI: 1.84–5.92) and playing/swimming in the lake (OR for everyday = 2.12, 95%CI: 1.00–4.51) (Table 4).

**Table 4:** Association between practice and *Schistosoma* infection (N = 424, *N = 212).

	Number of *S*. *mansoni* positive (%)	Crude Odds Ratio (95% CI)
Bathing at lake		
Never	22 (34.4)	1
Rarely	9 (27.3)	0.72 (0.28–1.80)
Sometimes	30 (31.6)	0.88 (0.45–1.73)
Everyday	135 (58.2)	2.66 (1.49–4.74)
Fetching water from lake		
Never	10 (33.3)	1
Rarely	15 (45.5)	1.67 (0.60–4.63)
Sometimes	47 (35.9)	1.12 (0.48–2.59)
Everyday	124 (53.9)	2.34 (1.05–5.22)
Washing clothes/utensils at lake		
Never	25 (32.1)	1
Rarely	14 (33.3)	1.06 (0.48–2.36)
Sometimes	73 (44.0)	1.68 (0.95–2.93)
Everyday	84 (60.9)	3.30 (1.84–5.92)
Playing (swimming) at lake*		
Never	20 (31.3)	1
Rarely	5 (23.8)	0.69 (0.22–2.14)
Sometimes	25 (33.8)	1.12 (0.55–2.30)
Everyday	26 (49.1)	2.12 (1.00–4.51)

2015 and 2016 data were integrated for risk factors analysis.

*Data is available only for 2016.

A positive association between bathing practice and attitude toward infection opportunity in daily life was also observed in the study (OR for everyday = 2.27, 95%CI: 1.37–3.77; OR for sometimes = 1.92, 95%CI: 1.09–3.40) (Table 5).

**Table 5:** Association between practice of bathing and attitude toward opportunity of infection with *Schistosoma* during daily life using ordered logistic regression modelling (N = 527).

	Agree to having opportunity of infection with schistosoma n (%)	Odds Ratio	Overall *p*-value
Bathing at lake			*p* = 0.0047
Never	26 (31.0)	1	
Rarely	15 (33.3)	1.18 (0.56–2.50)	
Sometimes	53 (44.9)	1.92 (1.09–3.40)	
Everyday	133 (47.5)	2.27 (1.37–3.77)	

Attitude was regrouped into the following three categories: agree (strongly agree + agree), neutral, and disagree (disagree + strongly agree).

### Education on schistosomiasis (contents of textbook)

In Kenya, an independent health subject was not included in the curriculum; thus, schistosomiasis was taught in the science subject. Pupils in class six learn about schistosomiasis in the science subject as “bilharzia”–a water-borne disease. Two kinds of textbooks published by different publishers were available in the study area, and their use depended on preference of the school. Both textbooks covered cause, transmission, and prevention. However, one of the textbooks wrongly described that drinking contaminated water is a mode of transmission, and the other missed the symptoms of intestinal schistosomiasis (S1 Table).

### Water use in primary schools

**Fig 2:**
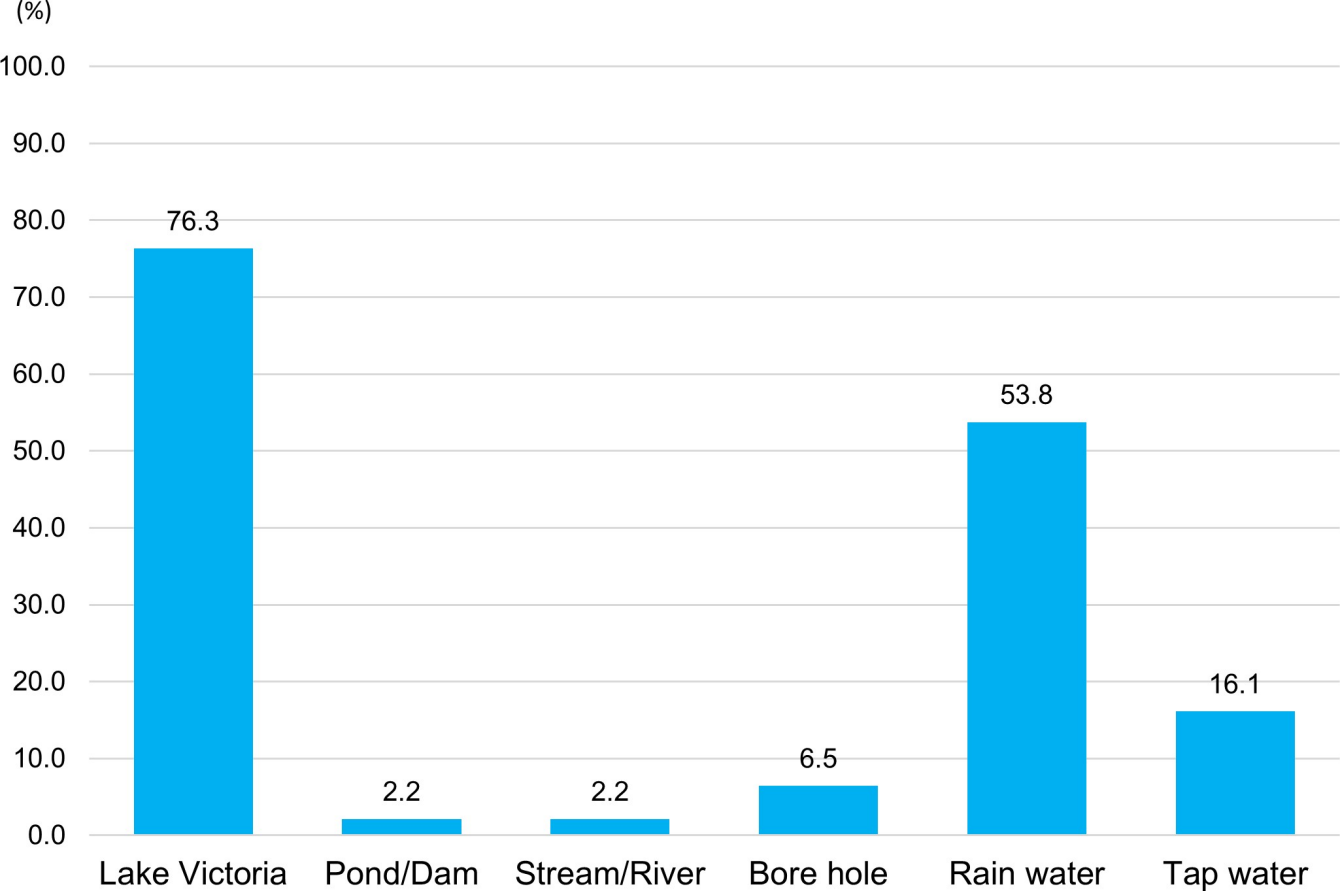
Water source of schools (Multiple answers, N = 93).

**Fig 3:**
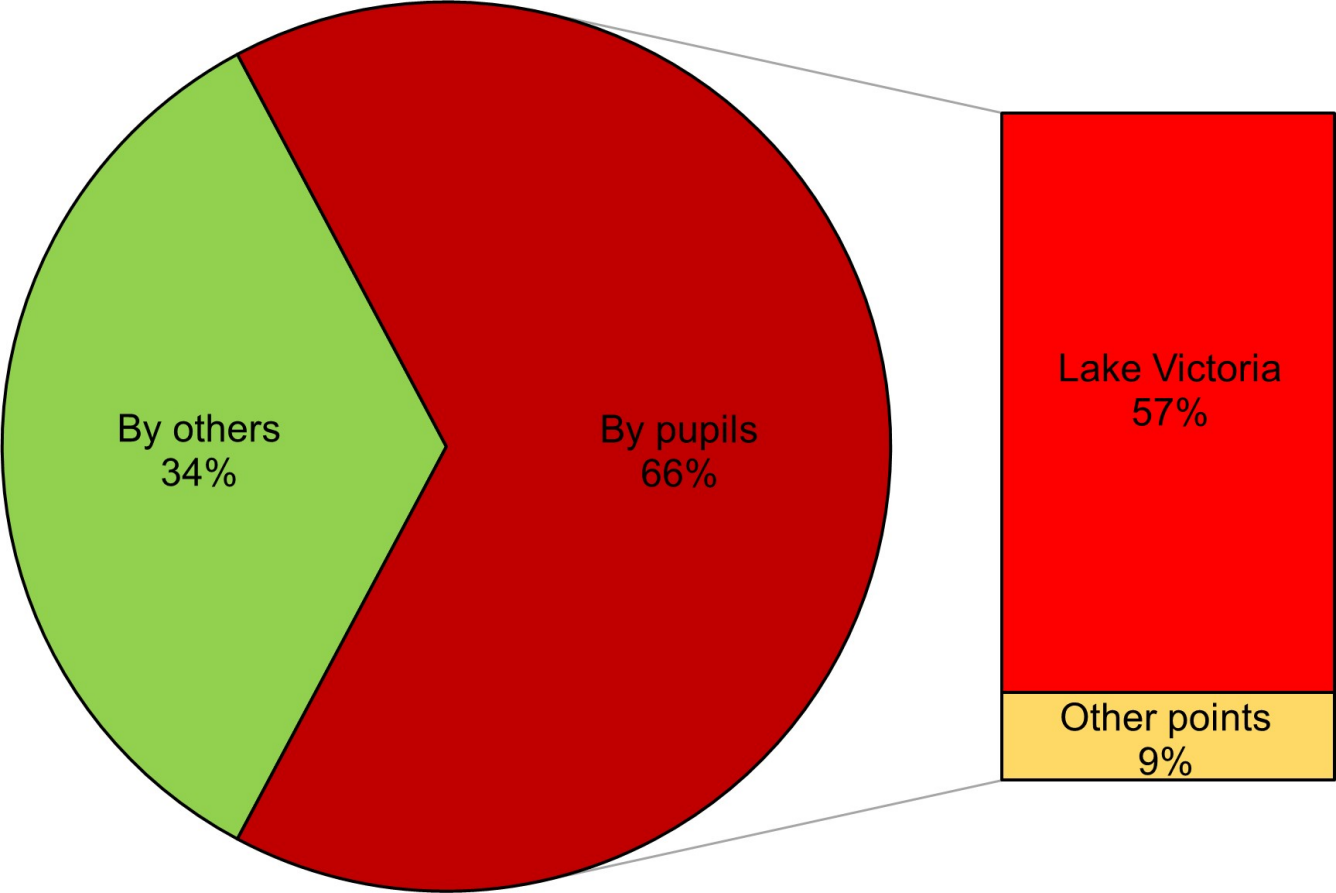
Proportion of schools using water fetched by pupils and fetching points (N = 93).

Of the 93 schools, 71 (76%) depended on Lake Victoria as a water source for daily school life (Fig 2). 54% of the schools had at least one rainwater harvesting tank and used rainwater during rainy season. 15 (16%) schools had tap water, but of those 11 schools used another water source in combination due to unstable tap water supply. 61 of 93 (66%) schools asked pupils to fetch/bring water for use in schools and of those, 53 schools had Lake Victoria as the nearest water source (Fig 3). The other eight schools either used water from a dam, stream, or a bore hole near the school. The main purposes of water use were for hand washing and drinking. Several schools also used water for school gardening purposes.

## Discussion

The prevalence of *S*. *mansoni* infection among pupils apparently changed in two years. It seemed to decrease; however, it has not decreased significantly when compared to the prevalence reported by previous studies in Mbita district [25–27]. Re-infection rate reported in previous studies in Kenya were 43.8%, 45 days after treatment, with a baseline prevalence of 84% [31], 34.4%, three to four months after treatment, with a baseline prevalence of 44.9% [32], and 8.6%, eight weeks after treatment, with a baseline prevalence of 47.4% [33]. Therefore, the 36% prevalence in the second year of the study, which was conducted six months after MDA with praziquantel, signifies that the re-infection rate in this area remained relatively high although cure rate after MDA was not examined.

In terms of knowledge about schistosomiasis, many pupils were able to choose correct answers for signs, transmission mode, and prevention methods. However, even though *S*. *haematobium* is rarely found in the study area [26], more than half of pupils chose “blood in the urine” as a sign of dominant schistosomiasis in Mbita. In Kenya, *S*. *haematobium* and *S*. *mansoni* exist, and both species can be found along the shores of Lake Victoria [4] except in Mbita area. Residents along the shore of Lake Victoria call schistosomiasis “*layo remo*” which means “urinating blood” in local language; therefore, there might be a misconception among the residents. However, it is unknown how popular the term is among children. Moreover, one of the science textbooks which was being used in this area only lists the sign of urinary schistosomiasis. This may also be one of the reasons why pupils chose “blood in urine” as a main sign of dominant schistosomiasis in Mbita. Previous studies in Tanzania [18] and Swaziland [17] also reported that signs of intestinal schistosomiasis were less known than urinary schistosomiasis among pupils, thus there might be a tendency that signs of *S*. *haematobium* are more known than that of *S*. *mansoni*.

In this study, the participants showed very good knowledge on schistosomiasis. This may be influenced by the Kenyan social and educational context. In Kenya, health is not a subject included in the primary education curriculum, but the Kenyan Ministry of Education encourages the inclusion of health topics in general subjects such as science, social studies, and language classes. The pupils learn about schistosomiasis in science class. Moreover, all pupils in Kenya who are supposed to graduate from primary schools must take a national examination, called the Kenya Certificate of Primary Education (KCPE), which affects their future education opportunity. Thus, they study hard, so it might be a reason why they have good knowledge of schistosomiasis because it was taught in classes. A study in Uganda [34] reported that health education using a child-friendly comic booklet failed to improve children’s knowledge. However, our study showed that even though special health education activities were not conducted, pupils can get useful information from general classes when both the method and the contents are appropriate to the context of each country or area. Moreover, the participants mentioned that the main source of information on schistosomiasis was teachers. It indicates that schools have a huge potential to distribute health information to the most susceptible age group as some previous studies reported [14,18]. Previous studies among adults have also reported that health workers, family, friends or neighbours are their source of information [15,16]. In the said studies, it is unknown which family member is the information source; however, other studies indicated that school children could pass health information to family and community members [35–37]. Therefore, appropriate health education in schools becomes very important and a useful tool for disease control.

Conflicting results in terms of attitude was found in the study. Nearly 75% of pupils thought that Lake Victoria is a source of schistosomiasis and half of pupils thought that they are not at risk of getting schistosomiasis. Yet, most of them come into contact with the lake water almost every day, as reflected by their answers to the questions on practices. Additionally, analyses of *Schistosoma* infection and practices at Lake Victoria showed a statistically positive association in accordance with previous studies conducted along the shores of Lake Victoria [26,38,39]. Although health education may succeed in giving correct information about schistosomiasis transmission, the demands of daily life and lack of safe alternatives seem to necessitate contact with the schistosome-infested water. Positive association between bathing in the lake and attitude on infection opportunity in daily life was also found, implying that pupils who bathe more frequently in the lake thought that they are more exposed to schistosomiasis than those who bathe less frequently in the lake. It also indicated that even though half of them did not agree that there is a possibility of them having a *Schistosoma* infection in daily life, they are aware that Lake Victoria is a source of infection and they go to the lake almost every day.

School children in the study area are taught in science class that one of the ways to prevent schistosomiasis is to avoid contact with contaminated water; however, most schools asked pupils to fetch/bring water from the lake for school life, which exposes them to infection. This contradiction occurred because there is no safe water source for schools and this challenge must be overcome for successful behaviour change. Some teachers said that they do not have any alternative to secure water, so they advised pupils to shorten the contact time with water as much as possible. Since free education is a government policy in Kenya, it is very difficult for schools to ask parents to contribute to provision of safe water. The school health project conducted by Nagasaki University and funded by JICA installed rainwater-harvesting tanks in all public schools that did not have the tank; however, water can be secured through this method only during the rainy season. Thus, efforts should be made to appeal to the government to install piped water or boreholes in schools. Moreover, a new innovative method to secure water is needed. Another challenge is toilet use. The rate of toilet use at home is clearly lower in comparison to toilet use in schools because some households do not have a toilet, thus children could not avoid open defecation. Moreover, open defecation is still common in this area and is not a disgraceful behaviour. Similar to a report from a different area in Kenya, this is a social and psychological challenge that must be overcome for behaviour change [23].

The current main preventive measure for schistosomiasis in Kenya is school-based mass drug administration; however, despite the government’s efforts, it has been intermittent due to logistical and financial challenges. In fact, school-based deworming was conducted four times in the study area during the past five years–in October 2012, June 2013, May 2015, and February 2016. However, praziquantel was not administered in May 2015, and although praziquantel was administered in the other years, its use was selective and very limited due to drug shortage. For example, during the MDA in February 2016, 88 (39%) out of 224 schools which conducted MDA used praziquantel in Mbita Sub-county. The keys for successful preventive chemotherapy (PCT) are continuous and more frequent drug administration [40,41]. There is a report that high prevalence of schistosomiasis was found after the withdrawal of school-based MDA [42].

The study also found that more than half of the pupils went to swim in the lake sometimes or every day. Thus, even if safe water will be provided, they might continue to go to the lake for recreation because of the hot weather in the area [43]. Moreover, fishing is the main source of income of residents in this area; hence fishermen are also at risk of schistosomiasis. The fishermen usually migrate from one fishing point to another for better catchment and this might make schistosomiasis control more complicated [44]. Since snails, which are the intermediate host, play an essential role in transmission [45], snail control is also important to prevent schistosomiasis [46].

This study has several limitations. First, urine samples were collected from each participant only once since *S*. *haematobium* is rare in the study area. Therefore, its prevalence might be underestimated. Second, the number of samples that were analysed for the prevalence of schistosomiasis became smaller than expected due to failure of some study participants to submit stool samples for three consecutive days. Third, since this study focused on school health, environmental factors such as distribution of snail species and *Schistosoma* infection among snails, which are important aspects for schistosomiasis control, were not documented.

This study confirmed that schistosomiasis is still highly prevalent among pupils in Mbita Sub-county, along the shore of Lake Victoria. Health education for pupils may be successful to acquire the correct knowledge; however, lack of safe water source and latrines blocked pupils’ behaviour change. From the school health viewpoint, health education that reflects the social and cultural context of the community in the contents and teaching methods are needed in order to improve their knowledge and behaviour and to change the social norm. In addition to this, regular mass drug administration with monitoring, provision of safe water, and encouragement of toilet construction and use in both schools and community/households in cooperation with WASH activities are needed. Furthermore, an environmental survey to control snails is also needed. Finally, a comprehensive and innovative approach which harmonises central and local governments and other stakeholders, as well as community members is important to prevent schistosomiasis in this area. If the educational message is feasible, behaviour will be changed and the situation among children will be improved.

## Supporting information

S1 Table Contents of science textbook on schistosomiasis. (DOCX)
