# Supplementary material for: Is there a gap between health education content and practice toward schistosomiasis prevention among schoolchildren along the shores of Lake Victoria in Kenya?
Source: PLoS Negl Trop Dis. 2019 Aug 19;13(8):e0007572. doi: 10.1371/journal.pntd.0007572 (PMC6715249; doi:10.1371/journal.pntd.0007572)
Supplement: S1 Table — (DOCX) [file pntd.0007572.s001.docx]

**S1 Table. Contents of science textbook on schistosomiasis**

|  | Textbook A | Textbook B |
| --- | --- | --- |
| Cause | Bilharzia worms | Flukes |
| Transmission | If one walks in, swims in or drinks contaminated water, the larvae enter his or her body through the skin, particularly the legs. | (All water-borne diseases)  Open sewer, Working barefoot in water-logged fields, Washing vehicles in the river |
| Signs and symptoms | - “Swimmer’s itch”. Itching in the bowels and the bladder | - Severe fever |
|  | - Skin rash if the worms are many. | - Itching may occur at the point of entry |
|  | - Fever after about four weeks. | - Blood in the urine |
|  | - A cough may develop |  |
|  | - Diarrhoea may develop. |  |
|  | - Blood in the urine. |  |
|  | - Blood in the stool (faeces). |  |
| Prevention | - Wear gumboots when walking in pools of water. | - Proper disposal of human waste. |
|  | - Put on gloves when working in water, for example, when transplanting rice seeding. | - Draining stagnant water and using chemicals to kill snails. |
|  | - Kill water snails using chemicals. | - Using protective boots and gloves. |
|  | - Always use a latrine or toilet to pass stool or urine. |  |
|  | - Drain stagnant water. |  |
|  | - Observe cleanliness at all times. |  |
